# BRET biosensors to study GPCR biology, pharmacology, and signal transduction

**DOI:** 10.3389/fendo.2012.00105

**Published:** 2012-08-29

**Authors:** Ali Salahpour, Stefano Espinoza, Bernard Masri, Vincent Lam, Larry S. Barak, Raul R. Gainetdinov

**Affiliations:** ^1^Department of Pharmacology and Toxicology, University of TorontoToronto, ON, Canada; ^2^Department of Neuroscience and Brain Technologies, Istituto Italiano di TecnologiaGenova, Italy; ^3^INSERM UMR 1037, Cancer Research Center of Toulouse and Université Paul SabatierToulouse, France; ^4^Department of Cell Biology, Duke UniversityDurham, NC, USA

**Keywords:** arrestins, GRKs, EPAC, cAMP, FRET, screening assay, TAAR1

## Abstract

Bioluminescence resonance energy transfer (BRET)-based biosensors have been extensively used over the last decade to study protein–protein interactions and intracellular signal transduction in living cells. In this review, we discuss the various BRET biosensors that have been developed to investigate biology, pharmacology, and signaling of G protein-coupled receptors (GPCRs). GPCRs form two distinct types of multiprotein signal transduction complexes based upon their inclusion of G proteins or β-arrestins that can be differentially affected by drugs that exhibit functional selectivity toward G protein or β-arrestin signaling. BRET has been especially adept at illuminating the dynamics of protein–protein interactions between receptors, G proteins, β-arrestins, and their many binding partners in living cells; as well as measuring the formation and accumulation of second messengers following receptor activation. Specifically, we discuss in detail the application of BRET to study dopamine and trace amine receptors signaling, presenting examples of an exchange protein activated by cAMP biosensor to measure cAMP, β-arrestin biosensors to determine β-arrestin recruitment to the receptor, and dopamine D2 receptor and trace amine-associated receptor 1 biosensors to investigate heterodimerization between them. As the biochemical spectrum of BRET biosensors expands, the number of signaling pathways that can be measured will concomitantly increase. This will be particularly useful for the evaluation of functional selectivity in which the real-time BRET capability to measure distinct signaling modalities will dramatically shorten the time to characterize new generation of biased drugs. These emerging approaches will further expand the growing application of BRET in the screening for novel pharmacologically active compounds.

## INTRODUCTION

Bioluminescence resonance energy transfer (BRET) is a process in which a non-radiative transfer of energy occurs between an excited luminescent enzyme/substrate donor complex and a fluorescent molecular acceptor that are separated by less than 100 Å. In many BRET studies published to date, the donors are variants of the enzyme, *Renilla reniformis* luciferase (Rluc), the enzymatically cleaved chemical substrate is coelenterazine, and the light emitting acceptors are variants of green fluorescent proteins (GFPs; Pfleger and Eidne, 2006). Degradation of a luminescent substrate by Rluc excites the GFP which in turn emits fluorescence. Resonance energy transfer (RET) techniques such as fluorescence resonance energy transfer (FRET) and BRET have become experimental techniques of choice for measuring constitutive and dynamic protein–protein interactions and interrogating changes in the activity of many biochemical signaling pathways, with FRET having the advantage of allowing cellular localization of the biological phenomenon that is studied. On the other side, BRET has advantage over FRET since it does not require an external illumination to initiate the energy transfer, which may lead to high background noise resulting from direct excitation of the acceptor or photobleaching. BRET experiments much like FRET can be conducted under conditions that more closely reflect the biochemical environments occurring in living organisms. As such, experimental platforms for BRET measurements have included model systems composed of bacteria, mammalian, and plant cells, and over 400 BRET related studies have been published since the first publication of Kai transcription factor interactions in bacteria (Xu et al., 1999). Over the last decade, the main usage of BRET has resided into investigating various protein–protein interactions (see Ayoub and Pfleger, 2010; Ferre et al., 2010; Lohse et al., 2012 for review) with major usage in the field of G protein-coupled receptors (GPCRs). However, more recently, several studies have applied BRET for the study of dynamic cellular processes, be it the modulation of the interaction of two proteins following a pharmacological treatment or the development of biosensors for various signaling pathways. In this review, we will discuss the development of some of these biosensors for the study of GPCRs. The term biosensor in this review will be applied to pharmacologically responsive interactions of GPCRs with other interacting proteins designed to study GPCR signaling pathways. We will however only briefly discuss the numerous studies that have reported pharmacologically evoked BRET variations on either homo or heteromeric GPCR complexes as those have in large part been reviewed elsewhere (Ayoub and Pfleger, 2010; Ferre et al., 2010; Lohse et al., 2012). Specifically, we will illustrate why BRET biosensors have become such an enabling technology for studying GPCR biochemistry *in cellulo* by presenting their use in characterizing the pharmacology and signaling of two important GPCRs implicated in monoamine transmission, the trace amine-associated receptor 1 (TAAR1) and dopamine D2 receptor (D2R).

## BRET BETWEEN RECEPTORS, G PROTEINS AND EFFECTORS

The first such study to utilize BRET to monitor the interaction between a GPCR and a G protein examined the interaction of β_2_-adrenergic receptor (β2AR) with G_αsβ1γ2_ (Gales et al., 2005). The β2AR was C-terminally tagged with Rluc, while the αs, β1 and γ2 were tagged with GFP10, a blue shifted variant of the GFP protein. Importantly, while the β1 and γ2 subunits were N-terminally tagged with GFP10, for the αs, the GFP10 was inserted between the helical and the GTPase domain of the protein in order to preserve αs functionality. Interestingly, a basal BRET signal indicative of constitutive interaction between all the tagged subunits and the receptor was observed. This BRET signal could be further enhanced with agonist stimulation when the heterotrimeric G protein was expressed, indicating either an increase in interaction between receptor and G proteins or a conformational change within the heterotrimeric complex. Since this first study, many others have been conducted on various receptor/G protein complexes, describing the kinetics, the orientation and the effects of ligands on the G protein–receptor interaction (Ayoub et al., 2007, 2009; Hasbi et al., 2007; Audet et al., 2008; Harikumar et al., 2008; Lohse et al., 2008; Kuravi et al., 2010; Oner et al., 2010; Busnelli et al., 2012).

Other BRET studies have investigated the interaction of GPCR with downstream effectors such as adenylyl cyclases or ion channels. Using a BRET approach, a constitutive interaction between β2AR–GFP and Kir3.1–Rluc potassium channel or adenylyl cyclase–Rluc was reported (Lavine et al., 2002). Importantly, the Kir3 BRET studies required the expression of a functional channel and therefore a significant BRET signal for Kir3.1–Rluc also required co-expression of the Kir3.2 or Kir3.4 subunits. Interestingly, agonist stimulation did not modulate these interactions, indicating that the complex between the receptor and these effectors did not dissociate during signal transduction.

## β-ARRESTIN-BASED BRET SENSORS

**FIGURE 1:**
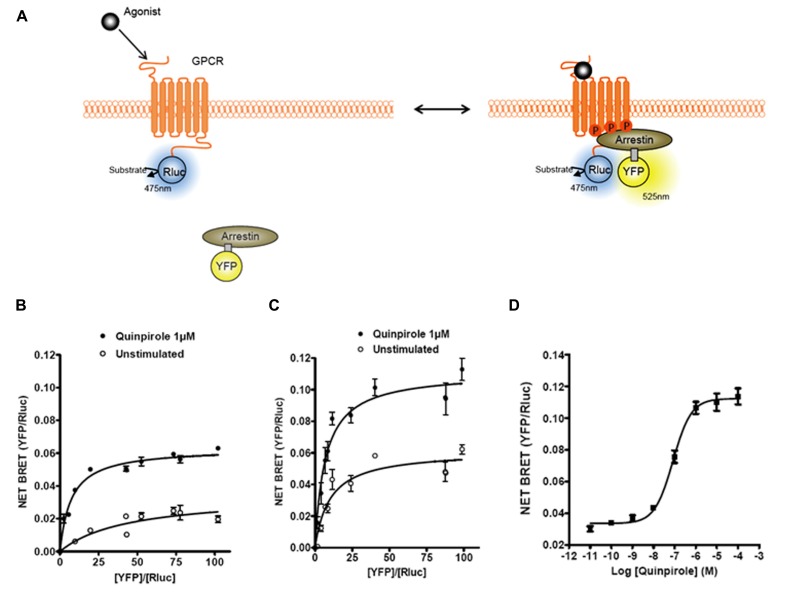
**Bioluminescence resonance energy transfer (BRET) monitoring of β-arrestins recruitment to activated GPCRs.** **(A)** Cartoon of postulated molecular events following activation of a GPCR by agonist that lead to recruitment of arrestin to phosphorylated receptor causing related BRET signal. Arrestins can be tagged with different versions of GFP (YFP, Citrine, or Venus; for review, see Pfleger and Eidne (2006)) and GPCRs are tagged with Rluc to monitor intermolecular interaction by BRET. **(B)** Titration curves of β-arrestin1–YFP recruitment to D2R–Rluc with or without D2R agonist quinpirole. **(C)** Titration curves on β-arrestin2–YFP recruitment to D2R–Rluc with or without D2R agonist quinpirole. **(D)** Dose–response curve of quinpirole effect on β-arrestin2 recruitment to D2R.

The first and most reported BRET-based sensor studies for GPCRs have been investigations into the dynamics of β-arrestin recruitment. Non-visual arrestins, β-arrestin1 (arrestin-2) and β-arrestin2 (arrestin-3), are cytosolic proteins that bind agonist stimulated receptors. In a pioneering study, Barak et al. (1997) showed that stimulation of β2AR with the full agonist isoproterenol resulted in the recruitment of GFP-tagged β-arrestin2 to the plasma membrane from a cytoplasmic localization. The recruitment of β-arrestin to a receptor is a signal event that initiates two important biological effects. First, β-arrestin recruitment leads to the internalization of the receptor into endocytic vesicles. Second, β-arrestin recruitment is associated with the stimulation of additional signal transduction pathways that are G protein independent (Lefkowitz and Shenoy, 2005). Over the years it has become clear that G protein- and β-arrestin-dependent signaling pathways may lead to different physiological effects. This has led to the idea that novel therapeutic approaches can be pursued based upon the selective modulation of either the G protein or the β-arrestin-dependent pathway (Whalen et al., 2011). As such, devising new approaches to interrogate β-arrestin signaling becomes an important avenue for drug development. Considering the many advantages of BRET, the measurement of β-arrestin recruitment to receptors using BRET became a complementary approach to the technique developed by Barak et al. (1997) that measures β-arrestin redistribution by analyzing high content images. The first study investigating β-arrestin recruitment to a GPCR using BRET employed a β-arrestin2–YFP molecule and β2AR–Rluc (Angers et al., 2000; see **Figure 1** for example and principle). Since this seminal study, β-arrestin recruitment has been reported in more than 30–40 manuscripts, where the time course, dose response, ligand dependency, and effects of receptor homo/hetero-oligomerization on recruitment have been assessed (for reviews, see Pfleger and Eidne, 2003; Pfleger et al., 2007). In addition, a β-arrestin2 recruitment approach by BRET was also successfully used in a high-throughput screening (HTS) platform to identify new antagonists of the chemokine CCR5 receptor (Hamdan et al., 2005), demonstrating the versatility of this assay.

In order to better understand the conformational changes incurred by β-arrestin2 upon its recruitment to the receptor, Charest et al. (2005) developed a β-arrestin2 biosensor termed double brilliance. The double brilliance is a single molecule biosensor in which β-arrestin2 is tagged with Rluc and YFP at the N and C terminus, respectively. The double brilliance can be used to monitor stimulation of GPCRs, since agonist stimulation of the receptors leads to an increase of the constitutive basal BRET signal of the double brilliance. Interestingly, GPCR stimulation does not always lead to an increase in the BRET signal of double brilliance. For angiotensin AT1 receptor signaling, it was observed that while stimulation with angiotensin increased the BRET signal, stimulation of the receptor with a β-arrestin-biased agonist (SII), produced a decrease in the BRET signal of double brilliance (Shukla et al., 2008). A similar observation was made for the parathyroid hormone receptor type 1 (PTH1R). Stimulation with PTH-(1–34) led to an increase in the BRET signal and treatment with (PTH-IA), a β-arrestin-biased ligand, resulted in a decrease in the BRET signal (Shukla et al., 2008). These observations indicate that β-arrestin can adopt multiple different conformations that are dependent on the ligand that stimulates the receptor, and that these changes can be probed using the double brilliance biosensor.

β-Arrestin2 is ubiquitinated following GPCRs activation and in the case of many receptors this process has been shown to be required for receptor endocytosis (Shenoy et al., 2001; Ahmed et al., 2011; Shenoy and Lefkowitz, 2011). Two major classes of GPCRs (class A and B) have been described with regard to the stability of their interaction with β-arrestins. Class A receptors form transient complexes, while class B receptors form stable complexes with β-arrestins after receptor stimulation. It has been shown that stable β-arrestin receptor complexes lead to sustained ubiquitination of β-arrestin while transient β-arrestin receptor complexes result in more temporary ubiquitination states (Shenoy and Lefkowitz, 2011). A BRET-based ubiquitination assay has been developed that allows the monitoring of β-arrestin ubiquitination in real time in live cells (Perroy et al., 2004). In these studies, β-arrestin2 was tagged with Rluc while the GFP was fused to ubiquitin. Agonist stimulation of both the β2AR, a class A receptor, and vasopressin V2 receptor (V2R), a class B receptor, results in a dose-dependent increase in BRET signal between Rluc–β-arrestin and GFP–ubiquitin, indicative of receptor-induced β-arrestin ubiquitination. Time course analysis of the BRET signal between β-arrestin and ubiquitin revealed that the signal was transient for β2AR stimulated samples while persistent for the V2R stimulated sample (Perroy et al., 2004). This observation is in agreement with prior experiments describing the dynamics of β-arrestin ubiquitination with class A and B receptors and demonstrates the utility of this sensor for studying the dynamics of β-arrestin ubiquitination in live cells.

Receptor interaction with G protein-coupled receptor kinases (GRKs) has also been investigated using BRET sensors. GRKs are a family of serine/threonine protein kinases that phosphorylate agonist stimulated receptors and for the most part desensitize their G protein signaling activity (Reiter and Lefkowitz, 2006). The first study utilizing BRET investigated the dynamics of an interaction between the Rluc–oxytocin receptor (Rluc–OTR) with GRK2–YFP (Hasbi et al., 2004). The authors showed that GRK2 is rapidly recruited to the receptor after agonist stimulation and that after several minutes of sustained stimulation the BRET signal decreases, consistent with the transient nature of the GRK/receptor interaction. Interestingly, the kinetics of GRK2 recruitment to OTR preceded β-arrestin2 recruitment to the same receptor suggesting that in this case, GRK2-mediated phosphorylation of OTR occurred before β-arrestin2 was recruited to the receptor. Other studies using BRET to investigate the interaction of GRKs with various GPCRs can be found in the following references (Huttenrauch et al., 2005; Small et al., 2006; Jorgensen et al., 2008, 2011; Namkung et al., 2009).

## PKA cAMP BRET BIOSENSORS

By coupling to either stimulatory Gs or inhibitory Gi protein pathways, many GPCRs modulate cAMP production, a second messenger that directly affects the function of many regulatory proteins. To study the dynamics of cAMP production, different BRET biosensors have been developed. The first BRET cAMP biosensor was based on protein kinase A (PKA; Prinz et al., 2006). PKA, a serine/threonine kinase, is composed of two regulatory and two catalytic subunits that dissociate upon binding of cAMP. There are two major PKA isoforms (I and II) that are expressed in mammalian cells with distinct biochemical and cellular functions. Prinz et al. (2006) created PKA fusion constructs by tagging the catalytic subunit with GFP2 (GFP-C) and regulatory subunits RI and RII with Rluc (RI–Rluc and RII–Rluc). Co-transfection of GFP-C subunit with either RI–Rluc or RII–Rluc results in a constitutive BRET signal that decreases in response to cAMP elevations from forskolin/IBMX treatment. However, only the RII–Rluc/GFP-C BRET sensor is sensitive to cAMP production by isoproterenol, a β2AR agonist, indicating that only PKA isoform II is in the correct spatial localization for detecting cAMP increases resulting from plasma membrane GPCR activation. However, when cells were treated with both isoproterenol and IBMX, both PKA isoform (RI and RII) were able to pick up the cAMP production induced by β2AR indicating that IBMX treatment alleviates the necessity for proper spatial localization to detect cAMP changes. Nevertheless, even in the presence of IBMX the magnitude of change in the BRET signal was greater from isoform II compared to isoform I, indicating that RII-based sensors may be more functionally sensitive and suitable for following GPCR-induced cAMP signals.

Although the PKA-based cAMP BRET biosensor is a powerful tool, it has an inherent caveat in that it is an intermolecular biosensor requiring the expression of two different proteins, i.e., regulatory and catalytic subunits. Therefore, a new generation of less complex single molecule cAMP BRET biosensors was developed from the guanine nucleotide exchange protein activated by cAMP (EPAC). To date two independent EPAC BRET biosensors have been described and used to study the modulation of the cAMP pathway by various GPCRs (Jiang et al., 2007; Barak et al., 2008).

## APPLICATION OF BRET BIOSENSORS FOR THE STUDY OF D2R AND TAAR1

The recent development of BRET and FRET biosensors has allowed the study of a variety of physiological processes, such as formation of second messengers, protein kinases activity, protein–protein interactions, and protein trafficking (Vilardaga et al., 2003; Ni et al., 2006; Ayoub and Pfleger, 2010; Milligan, 2010; Lohse et al., 2012). With the application of different biosensors to the same receptor it is also possible to monitor the multidimensional complexity of signaling of a given receptor under the same experimental conditions. For example, the D2 dopamine receptor (D2R) is a Gi coupled receptor that is known to decrease cAMP levels upon stimulation by an agonist (Beaulieu and Gainetdinov, 2011). Recently, a new modality of G protein-independent β-arrestin2-mediated signaling has been described for D2R (Beaulieu et al., 2007; Beaulieu and Gainetdinov, 2011). Both G protein-dependent and -independent signaling pathways play important roles in dopamine-related physiological and pathological processes (Beaulieu et al., 2005, 2007; Beaulieu and Gainetdinov, 2011). Using a heterologous expression system and two different BRET biosensors, we assessed cAMP signaling and β-arrestin2 recruitment following activation or blockade of D2R (Masri et al., 2008). In another set of studies aimed at better understanding of monoamine transmission, we used BRET to study the signaling properties and pharmacology of TAAR1. In recent years, the TAAR1 has attracted attention as a potential new target for the modulation of the dopaminergic system (Lindemann et al., 2008; Sotnikova et al., 2009; Revel et al., 2011). Using BRET assays, we have evaluated the ability of TAAR1 and D2R to form a functional heterodimer and alter each other’s signaling and functions (Espinoza et al., 2011).

## USE OF EPAC cAMP BRET BIOSENSOR TO STUDY D2R AND TAAR1

**FIGURE 2:**
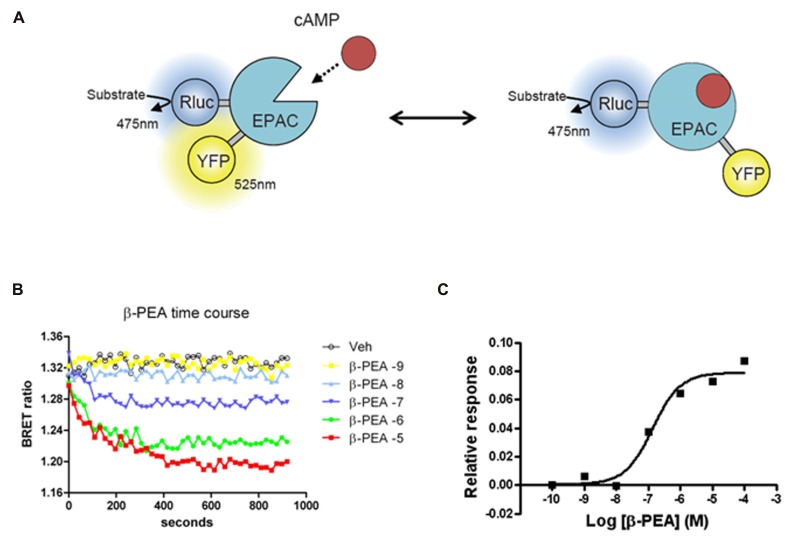
**Bioluminescence resonance energy transfer (BRET) EPAC biosensor for cAMP monitoring.** **(A)** Cartoon of the postulated molecular rearrangement of the full-length EPAC protein with and without intracellular cAMP increase causing related BRET signal. EPAC, a protein that changes conformation upon cAMP binding, was tagged with a BRET donor (Rluc) and a BRET acceptor (YFP) on each extremity of the protein to monitor intramolecular BRET signal. **(B)** Time course effects β-PEA in cells transiently transfected with EPAC and TAAR1. BRET ratio is measured as YFP/Rluc ratio and the readings are started right after β-PEA addition. Cells are exposed to different concentration of β-PEA (from 1 nM to 100 μ) or control medium. The decrease in BRET ratio indicates an increase in cAMP concentration. β-PEA induces a robust increase in cAMP level that lasts along the duration of the entire experiment (20 min). **(C)** Dose–response of β-PEA effect on TAAR1-dependent cAMP accumulation after 10 min of stimulation.

The cAMP EPAC biosensor used in our studies was originally developed as a FRET biosensor (DiPilato et al., 2004) and later adapted for BRET applications (Barak et al., 2008). In this sensor, both the donor and the acceptor are located within the same protein leading to an intramolecular energy transfer. The sensor is made of EPAC, a protein that changes conformation upon binding cAMP. The premise behind the original FRET biosensor was to tag the full-length protein by a donor (enhanced cyan fluorescent protein, ECFP) and an acceptor (Citrine) on each extremity of the protein (see **Figure 2** for example and principle). The sensor was further improved by using a truncated form of the protein, comprised only of the cAMP binding domain and named ICUE2. ICUE2 was transformed in a BRET sensor by replacing the ECFP with the Rluc (Barak et al., 2008). At resting levels, there is considerable basal energy transfer between Rluc and Citrine resulting in a high BRET ratio, suggesting that in the absence of cAMP the donor and the acceptor are in close proximity. As cAMP increases, BRET ratios decrease, presumably due to a conformational change leading to increased distance between the Rluc and Citrine. As for other BRET sensors, the main advantage of EPAC is the possibility to measure the fluctuations of cAMP in real time and so to evaluate the contribution and the kinetic of different systems that modulate cAMP levels. GPCRs that couple to Gs or Gi are thus suitable targets for investigations with this sensor. Importantly, the BRET response with this sensor is reversible since the removal of the agonist or the addition of an antagonist results in a decrease of the response (DiPilato et al., 2004). Stimulation of both TAAR1 and β2AR leads to increases in cAMP levels readily measurable with the EPAC biosensor (Barak et al., 2008; Violin et al., 2008). But the degree of desensitization of these two receptors is different, being stronger for the β2AR. In comparison to β2AR, over-expressed TAAR1 has relatively minor level of plasma membrane expression, having predominantly an intracellular localization. Due to either poor surface expression or the fact that TAAR1 may be less prone to desensitization, the over-expression of β-arrestin2 has substantially less of an effect on the kinetic of cAMP response of TAAR1 in comparison to β2AR (Barak et al., 2008).

Considering the simplicity of BRET experimental procedures it is evident that the EPAC sensor is a suitable tool for medium/HTS for identification of ligands of GPCRs that modulate cAMP. For example, we tested many potentially active compounds for their activity at membrane-expressed human TAAR1, confirming activity of some of the already known ligands and discovering interesting new ligands (Barak et al., 2008). In a follow-up study, we used EPAC sensor to screen for novel TAAR1 ligands against a limited library containing 1,000 drug-like compounds (data not published). About 20 potentially active compounds were initially identified but after excluding compounds that showed activity in cells expressing only the EPAC, four were confirmed as putative human TAAR1 agonists. Three of these compounds were known agonists of TAAR1, tyramine, 3-methoxytyramine, and 4-methoxytyramine, thereby validating specificity and sensitivity of this screening approach. Interestingly, the fourth compound was guanabenz that was later identified, by another group, as one of the most potent agonists of TAAR1 (Hu et al., 2009). In general, comparing with a standard radioactive cAMP column chromatography assay, the BRET method was found to be qualitatively similar but slightly more sensitive.

In another study, we used the EPAC biosensor to study the functional interaction between TAAR1 and D2R and observed that blockade of D2R selectively enhances TAAR1 signaling indicating a functional interaction between these two receptors (Espinoza et al., 2011). We have also used the EPAC sensor to evaluate the ability of different antipsychotics to block D2R-mediated signaling (Masri et al., 2008). Almost all antipsychotics showed an intrinsic activity as inverse agonists, with the exception of aripiprazole, a known partial D2R agonist. However, against quinpirole, a selective D2R agonist, the compounds blocked the response with different potencies and affinities, while aripiprazole’s maximum efficacy of blockade was only 30%. All these are examples of how the EPAC sensor is a powerful tool for studying cAMP fluctuations in living cells.

## β-ARRESTIN2 RECRUITMENT STUDIES FOR D2R

β-Arrestins were originally identified for their role in desensitizing GPCRs while subsequent studies have shown that β-arrestins can also activate signaling cascades independently of G proteins by acting as multifunctional scaffolding proteins (for review: Shenoy and Lefkowitz, 2011). Therefore, to fully characterize the mode of action of GPCR ligands, it is useful to evaluate their effects on both the G protein-mediated pathway and the β-arrestin pathway. A study conducted using β-arrestin2 knockout mice demonstrated that D2R can engage the Akt/glycogen synthase kinase 3 (GSK-3) signaling pathway by a mechanism that involves a signaling complex comprised of β-arrestin2, Akt, and the multimeric protein phosphatase PP2A (Beaulieu et al., 2005). This signaling pathway is essential since it is involved in the expression of some dopamine associated behaviors that are sensitive to antipsychotic drugs (Beaulieu et al., 2004). In fact, the clinical efficacy of almost all antipsychotic drugs (typical and atypical) is directly correlated with their binding affinity to D2R and their capacity to antagonize this receptor (Creese et al., 1976). The activity of these compounds has been extensively studied for cAMP signaling but little is known about ligand selectivity for β-arrestin-mediated signaling pathways. As described above, using BRET, it is possible to monitor real-time translocation of β-arrestin to an activated GPCR as well as to measure the effective dose (EC50) of an agonist or the inhibitory concentration (IC50) of different antagonists and compare their activity on this signaling pathway.

We used the β-arrestin and the EPAC BRET biosensors to define more precisely how antipsychotics affect dopamine D2 receptor signaling. While antipsychotics had antagonistic properties with very variable efficacies and potencies regarding Gi/o-mediated cAMP production, all these molecules were highly effective at antagonizing β-arrestin2 translocation to D2R with potencies between 3- and 150-fold higher than at the G protein-mediated pathway (Masri et al., 2008). These results suggested that clinically effective antipsychotics may act as preferential antagonists for D2R/β-arrestin2-mediated signaling rather than Gi/o-mediated signaling by this receptor. It is tempting to speculate that antipsychotics may exert their therapeutic effects in part by blocking β-arrestin2-mediated D2R signaling while inducing some of their side effects through modulation of other pathways. This idea was recently explored by using analogs of the atypical antipsychotic aripiprazole. These functionally selective β-arrestin2-biased D2R ligands exhibit antipsychotic activity *in vivo* and do not induce catalepsy in wild type mice (Allen et al., 2011). Thus, biased pharmacological approaches aimed at selectively targeting the β-arrestin2 signaling pathway activated by dopamine D2R may provide safer and more effective antipsychotics and protect against some of the motor side effects associated with this class of drugs.

## D2R AND TAAR1 HETERODIMERIZATION

Direct protein–protein interaction is a commonly accepted concept in cell biology and in the recent years the possibility of homo- or heterodimerization of GPCRs has been fully appreciated. Using different techniques, it has been shown that many GPCRs exist as homo-, heterodimers or even as oligomers and techniques such as FRET and BRET have strongly contributed to the characterization of these physiological phenomena. The interaction between two or more GPCRs can alter important functions of these receptors such as cell surface delivery, G protein coupling and pharmacology, to name a few (Dalrymple et al., 2008; Milligan, 2010).

**FIGURE 3:**
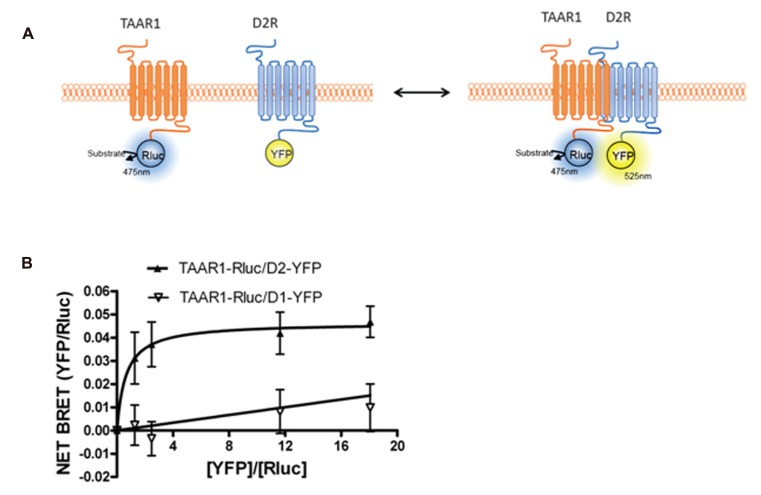
**Bioluminescence resonance energy transfer (BRET) monitoring of TAAR1 and D2R heterodimerization.** **(A)** Cartoon of postulated molecular events during heterodimerization of GPCRs causing related BRET signal. TAAR1 is tagged with Rluc and D2R is tagged with YFP to monitor intermolecular interaction between these two receptors. **(B)** BRET titration curve of physical interaction between TAAR1–Rluc and D2R–YFP. A fixed amount of TAAR1–Rluc and increasing amount of D2R–YFP were co-expressed in the same cells. BRET was measured 20 min after the addition of the substrate, coelenterazine h. To test specificity of BRET signal between TAAR1 and D2R, BRET was also measured between TAAR1–Rluc and increasing amount of D1R–YFP. The hyperbolic shape of the curve indicates that TAAR1–Rluc and D2R–YFP form a constitutive heterodimer when co-expressed in the same cells. A linear increase in the BRET signal is observed between TAAR1–Rluc and D1–YFP indicating a non-specific, bystander BRET between these receptors.

Intriguingly, when TAAR1 and D2R were co-expressed in the same cells, TAAR1-mediated cAMP signaling was increased when D2R receptors were blocked with the antagonist haloperidol (Espinoza et al., 2011). This signaling enhancement was selective for TAAR1 and did not occur with other Gs-coupled receptors. One possibility that we explored was heterodimerization between TAAR1 and D2R. Using BRET, we observed that TAAR1 and D2R can indeed exist as heterodimers when co-expressed in HEK-293 cells (Espinoza et al., 2011). Since BRET, like other techniques, has certain limitations, it was important to have appropriate controls to verify the reliability of the interaction (Marullo and Bouvier, 2007). We therefore performed BRET titration assays with a constant amount of the donor fusion protein (TAAR1–Rluc) and an increasing amount of the acceptor fusion protein (D2–YFP). The hyperbolic curve obtained for TAAR1–Rluc and D2–YFP, but not for D1–YFP, confirmed the selectivity of the interaction between these two receptors (see **Figure 3**). Furthermore, in a BRET competition assay, where an equivalent quantity of untagged D2R was co-expressed with TAAR1–Rluc and D2–YFP, the BRET signal was significantly decreased, while expression of untagged D1R had no effect on the BRET. Using a whole cell ELISA, we also showed that TAAR1 and D2R can co-internalize upon agonist stimulation of D2R, further confirming the physical interaction between the two receptors. Using cellular fractionation, we further noted that the heterodimer was mainly expressed at the plasma membrane and that treatment with D2R antagonist haloperidol could almost completely abolish the BRET signal from the heterodimer. These results suggest that haloperidol treatment either leads to the disassembly of the dimer or that this treatment induces a conformational change such that the distance between the donor and acceptor fluorophores are increased to the point where there is a decline in energy transfer. Notably, ligand-promoted BRET changes have been reported for other GPCR homo/heterodimers as well (Dalrymple et al., 2008; Milligan, 2009). Finally, we have observed some functional consequences of the putative TAAR1–D2R interaction where in TAAR1-KO animals, haloperidol-induced striatal c-Fos expression and cataleptic responses were significantly reduced (Espinoza et al., 2011).

## CONCLUSION AND PERSPECTIVES

Bioluminescence resonance energy transfer biosensors have been instrumental in advancing our understanding of GPCR signal transduction by providing optical tools to study real-time interactions between receptors, the recruitment of binding partners to receptors, and variations in concentrations of second messengers generated downstream of receptors. Most importantly, BRET studies are conducted in live cells and enable the study of a wide variety of signaling systems to be probed under biologically relevant conditions, with minimal perturbation and in a quantitative manner. GPCR-related BRET biosensors have already established utility as screening platforms in drug discovery process. At the same time, the number of BRET sensors described to date represents only a small fraction of their potential applications in biology and the list of new sensors to interrogate various signaling pathways and protein–protein interactions is rapidly growing. In particular, we anticipate that a new generation of BRET sensors will enable us to probe GPCR signaling in much greater detail than currently possible; and potentially lead us to new classes of “biased” ligands with unique therapeutic profiles (Beaulieu et al., 2005, 2007; Masri et al., 2008; Beaulieu and Gainetdinov, 2011; Whalen et al., 2011)

## Conflict of Interest Statement

The authors declare that the research was conducted in the absence of any commercial or financial relationships that could be construed as a potential conflict of interest.
